# Effects of Heat Treatment on Color, Dimensional Stability, Hygroscopicity and Chemical Structure of Afrormosia and Newtonia Wood: A Comparative Study of Air and Palm Oil Medium

**DOI:** 10.3390/polym15030774

**Published:** 2023-02-02

**Authors:** Lionnel Frederique Bidzanga Bessala, Jingjing Gao, Zhengbin He, Zhenyu Wang, Songlin Yi

**Affiliations:** Beijing Key Laboratory of Wood Science and Engineering, MOE Key Laboratory of Wooden Material Science and Application, Beijing Forestry University, Beijing 100083, China

**Keywords:** Afrormosia, Newtonia, heat treatment, palm oil, dimensional stability, hygroscopicity, chemical structure

## Abstract

In recent years, China is increasingly dependent on imported wood. Afrormosia and Newtonia are some of the imported species with good utilization potential. However, both of them also have problems with poor dimensional stability. In order to make better use of these two types of wood, the influence of heat treatment under air and palm oil conditions on the color, dimensional stability, and hygroscopicity of Afrormosia and Newtonia was investigated. The Afrormosia and Newtonia wood samples were heated in air or palm oil medium for two hours at 160 °C, 180 °C and 200 °C, respectively. Then, the color, weight changes, swelling, moisture absorption and chemical structure were evaluated for each case. As results, the heat treatments with air or palm oil increased the dark color of Newtonia and Afrormosia wood and this increase was proportional to the treatment temperature. The tangential and radial swelling coefficient for air heat treatment of Afrormosia wood at 200 °C were, respectively, reduced by 24.59% and 19.58%, while this reduction for Newtonia was 21.32% and 14.80%. The heat treatment in palm oil further improved the stability and hygroscopicity of the wood, showing that the Afrormosia samples treated by palm oil at 200 °C underwent a decrease of its tangential and radial swelling coefficient, respectively, by 49.34% and 45.88%, whereas the tangential and radial swelling coefficient of Newtonia treated under the same conditions were reduced by 42.85% and 33.63%, respectively. The heat treatments of Afrormosia and Newtonia samples under air at 200 °C diminished the water absorption by 21.67% and 22.12%. The water absorption of Afrormosia and Newtonia heat-treated under palm oil at 200 °C was reduced, respectively, by 39.40% and 37.49%. Moreover, the FTIR analysis showed the decrease of hydroxyl groups in proportion to the wood treatment temperature.

## 1. Introduction

Afrormosia (*Pericopsis elata* Van Meeuwen) is an angiosperm belonging to the Fabaceae family. Its wood is yellow-brown in color with a fine grain and a slight interlock. It is a kind of wood that dries slowly and shows little deformation after drying [1]. As for Newtonia (*Newtonia paucijuga* Harms), it also belongs to the Fabaceae family. Its heartwood is pinkish brown. The yarn is wavy and the grain is fine to medium coarse. The wood is moderately heavy and moderately durable, showing moderate resistance to termite attack but susceptible to beetle and marine borer attack [2]. It is therefore clear that Afrormosia and Newtonia woods have good physical and aesthetic properties, which explains why they are used in several sectors of activity, such as carpentry and cabinet making. However, like almost all woods, they have shortcomings related to their low durability, dimensional instability and high equilibrium moisture content. Many methods have been developed to correct this kind of defect, in particular, chemical treatment [3,4,5,6,7,8,9,10,11,12,13,14], mechanical treatment [15], magnetic treatment [4] and heat treatment [16,17].

Speaking of heat treatment, it has the highest market conversion rate and broad future prospects in the functional technology of wood [18], which is why this treatment has been the subject of numerous studies. Indeed, studies on certain wood species have shown that heat treatment of wood with steam reduces swelling and shrinkage and lowers its equilibrium moisture content [19]. Aydemir [20] investigated the effects of heat treatment on the dimensional stability of hornbeam and uludag fir wood and concluded that water absorption and swelling were lower for heat-treated wood samples and that it depended on the wood species, temperature, and treatment time. Conclusions shared by Aytin [21] studying the effects of heat treatment with the ThermoWood^®^ method on the equilibrium moisture content and dimensional stability of wild cherry wood came to the same conclusions as previously presented by Aydemir. Bekhta [22] estimated that a heat treatment at 200 °C for 24 h stabilizes the wood better than a treatment at 200 °C for 2 h. Furthermore, he asserted that swelling, both in tangential and radial directions, decreased with increases in treatment duration. For Dubey’s [23] opinion, heat treatments aimed at stabilizing the dimensions of the wood, must be executed at a minimum temperature of 160 °C.

Research has also been conducted on heat treatments using heat transfer fluids, such as certain oils [24,25,26,27] and air. For example, Priadi [28] kiln-dried the jabon, sengon and mangium wood, then subjected them to heat treatment under air at temperatures of 120 °C, 150 °C, and 180 °C for two and six hours. The results showed that the heat treatments under air reduced water absorption and significantly improved dimensional stability of the samples. With regard to studies relating to the treatment of wood under oil, Dubey [23,25], in studying the influence of thermal treatment with linseed oil on the hygrometric properties of *Pinus radiata*, observed an improvement in the percentage of volumetric swelling proportional to the treatment temperature. Wang [27] found that palm oil is more effective than soybean oil in improving the dimensional stability of oil heat treated white spruce. Cheng [29] states that vegetable oils have a high calorific value, which allows them to transfer heat more uniformly during heat treatment. Moreover, vegetable oils generally have a higher boiling point than that required to heat treat wood, making them suitable for such use. Lee [26] reviewed the literature on thermal treatment of wood with oil and stated that: The type of oil, duration of heating, peak temperature, and wood species are the most important parameters of thermal modification processes of wood, especially when improving wood dimensional stability. He goes further to state that vegetable oils in particular palm oil, have low environmental toxicity and offer the possibility of adding additives during heat treatment to improve the properties of the wood. Indeed, palm oil occupies only 6% of the area used for the cultivation of vegetable oils but produces more than a third of the world’s vegetable oil [30] with an estimated production in 2020 of 73 million tons, making palm oil the most produced vegetable oil in the world [31] of which only 80% is consumed as food [32]; moreover, this production is expected to double by 2030 [31]. It is therefore a highly available oil whose cultivation has a reduced impact on deforestation compared to other vegetable oils such as soybean oil, whose cultivation occupies 40% of the world’s oilseed crop area and produces only 22% of the world’s vegetable oils [30].

In addition, some authors have attempted to explain, from a chemical point of view, the mechanisms that justify the dimensional stabilization of wood by heat treatment, and high temperatures have been found to reduce the number of hydroxyl groups in the cell wall, while increasing the crystallinity of cellulose [20,33]. This leads to a decrease in the exchange between water and wood, thus improving the dimensional stability of wood while influencing the color of the wood [11,34,35].

Heat treatment is very effective to stabilize the hygrometric properties and increase the natural durability of wood. However, intuitive observations allow for noticing that heat-treated wood, in general, undergoes a change of color, which has significant impacts on its utilization. For this reason, research has been carried out on this subject. Studies on the color change of wood after heat treatment are generally based on the CIE standard color measurement method specified by the International Commission on Illumination (CIE); it is a system similar to Descartes XYZ system where XYZ are, respectively, replaced by the yellow-blue index (b*), the red-green index (a*) and the lightness index (L*). Torniainen [36] suggested that L* should be the control parameter for assessing the quality of heat-treated wood, and that a* and b* should only be used as secondary parameters of analysis. Thus, several authors have used these indices to characterize color changes in so-called temperate gymnosperm wood species [22,33,35,37]. As a result, they have shown that wood color change upon heat treatment correlates with lignin and hemicellulose degradation and extractives accumulation [38]. The intensity of color change during heat treatment of wood is a function of wood density, heat treatment duration and temperature, and these variables are sufficient to predict color changes of heat-treated wood [39]. Regarding tropical wood or angiosperms, we can cite the studies of Ayata [40,41], who measured the effect of heat treatment on the brightness and color of Afrormosia (*Pericopsis elata*), Doussie (*Afzelia bipindensis*), Frake (*Terminalia superba*) and Iroko (*Chlorophora excelsa*) wood. The results showed that color (L*, a* and b*) and gloss (parallel and perpendicular to the grain at angles of 20°, 60° and 85°) were modified by the treatment. In fact, L* and surface glossiness decreased and ΔE* and a* values increased with treatment intensity. Furthermore, Dubey [25] showed that the oil, once impregnated in the wood, prevents it from oxidizing and thus improves its color stability.

Afrormosia and Newtonia are two wood species of African origin and largely imported by China. Undoubtedly, heat treatment can be one of the most effective methods to improve their quality. However, to our knowledge, there are almost no articles relating to the comparative study of the effects of heat treatments under air and palm oil conditions on certain physical properties of these two species, in particular, color, weight variation, swelling and water absorption. Consequently, the objective of the present study is to demonstrate the differences and mechanism of Afrormosia and Newtonia heat treatment in air and palm oil medium, which will further optimize the heat treatment process and expand the utilization of Afrormosia and Newtonia wood.

## 2. Materials and Methods

### 2.1. Sample Preparation

The Afrormosia (*Pericopsis elata* Van Meeuwen) and Newtonia (*Newtonia paucijuga* Harms), of African origin, that we used to make the experiment samples were collected from the Landbond Group of China. The dimensions of the samples were 20 mm × 20 mm × 20 mm (L × T × R) with an initial moisture content of approximately 60%. All specimens were dried in an oven at 103 ± 2 °C to absolute-dried condition before heat treatment.

### 2.2. Heat Treatment

The heat treatment of wood samples was performed in a homemade sealed heat treatment tank. During the heat treatment, the tank was filled with air or palm oil. In order to avoid significant heat loss, thermal insulation panels were used. Both Afromosia and Newtonia samples were heat treated in air or palm oil medium for 2 h. The treatment temperature was set at 160 °C, 180 °C and 200 °C, respectively, which was monitored by temperature sensor. All samples were divided into 16 groups of 7 samples each. The labelling of each sample group is shown in Table 1.

### 2.3. Color Change

Specimens were passed to the colorimeter in order to measure the parameters L*, a*, and b*. The color change of the heat-treated samples was measured by a Shenzhen 3NH NR200 colorimeter (NR200, 3NH, Shenzhen, China) according to the CIELab system. ΔE* was calculated according to Equations (1)–(4).

(1) ΔE*=ΔE*2+ΔE*2+ΔE*212

(2) $$\Delta L^{*}=L^{*}{}_{\mathrm{heat}-\mathrm{treated}}-L^{*}{}_{\mathrm{untreated}}$$

(3) $$\Delta a^{*}=a^{*}{}_{\mathrm{heat}-\mathrm{treated}}-a^{*}{}_{\mathrm{untreated}}$$

(4) $$\Delta b^{*}=b^{*}{}_{\mathrm{heat}-\mathrm{treated}}-b^{*}{}_{\mathrm{untreated}}$$

### 2.4. Weight Change

After the heat treatment, the weight of samples generally reduces due to the thermal-induced degradation of wood components. However, the impregnation of liquid into the wood during palm oil heat treatment may cause the increase of sample weight, to some degree. The density of samples after heat treatment was evaluated based on Equation (5). The weight loss or weight gain was also evaluated by comparing the mass of the samples before and after the heat treatment under air or palm oil conditions. The calculation was made using Equations (6) and (7) as below.

(5) $$\rho_{t}=\frac{W_{t}}{V_{t}}$$

(6) $$\mathrm{WL}\left(\%\right)=\frac{W_{u}-W_{t}}{W_{u}}\times 100$$

(7) $$\mathrm{WG}\left(\%\right)=\frac{W_{t}-W_{u}}{W_{u}}\times 100$$

where $V_{t}$, $W_{u}$ and $W_{t}$ are, respectively, the volume of sample after heat treatment and the weight before and after heat treatment; WL is the weight loss in percentage, $\rho_{t}$ (g/cm^3^) is the density of the treated wood and WG is the weight gain in percentage.

### 2.5. Swelling Coefficients and Anti-Swelling Efficiency

After heat treatment, the longitudinal, radial, and tangential dimensions of these samples were measured using a caliper with an accuracy of ±0.01 mm. The volumes were calculated by taking the product of the respective values found. Then, the samples were stored at a sealed container with a temperature of 20 ± 2 °C and a humidity of 65 ± 3% until the volume became constant. Subsequently, the dimension and volume of swelled samples were determined [42]. This allowed the calculation of linear swelling coefficients (S_l_), volumetric swelling coefficient (S) and anti-swelling efficiency (ASE) using Equations (8)–(10), respectively.

(8) $$S_{l}=\frac{l_{w}-l_{0}}{l_{0}}\times 100$$

where $l_{w}$ is the tangential or radial dimension of swelling sample after water absorption, $l_{0}$ is the tangential or radial dimension of the oven-dried sample, and $S_{l}\left(\%\right)$ is the linear swelling coefficient in percentage.

(9) $$S\left(\%\right)=\frac{V_{S}-V_{0}}{V_{0}}\times 100)$$

where $V_{S}$ is the volume of swelling sample and $V_{0}$ is the volume of the oven-dried sample; $S\left(\%\right)$ is the volumetric swelling coefficient in percentage.

(10) $$\mathrm{ASE}\left(\%\right)=\frac{S_{u}-S_{t}}{S_{u}}\times 100$$

where $S_{u}$ and $S_{t}$ are the swelling coefficients of untreated and treated wood, respectively.

### 2.6. Moisture Absorption (MA)

Moisture absorption was determined by putting specimens in the same chamber as swelling experiment with a constant temperature of 20 ± 2 °C and a humidity of 65 ± 3% to reach the equilibrium moisture content according to the GB/T 1931–2009 standard [43].

(11) $$\mathrm{MA}=\frac{w_{a}-w_{0}}{w_{0}}\times 100$$

where $w_{a}\;$and $w_{0}\;$are, respectively, weight of the specimens after conditioning (g) and weight of oven dried specimens (g).

### 2.7. FTIR

Fourier transform infrared spectroscopy (FTIR) was performed with an ATR-FTIR Nicolet 6700 spectrometer (Thermo Fisher Scientific, Waltham, MA, USA). The dimensions of the samples were nearly 10 mm × 10 mm × 0.6 mm. A total of 32 scans were performed for each sample and were recorded in the range of 4000 to 400 cm^−1^ with a resolution of 4 cm^−1^ in transmission mode.

### 2.8. Statistical Analyses

The statistical analyses were completed using IBM SPSS Statistics 21 software. The means of the parameters of each batch was calculated and compared using the Student’s t-test for paired samples with a percentage of the confidence interval equal to 95% in order to ensure that the heat treatment has a significant influence on the studied parameters of the wood. Otherwise, the difference in means was considered to be zero.

## 3. Results and Discussion

### 3.1. Color Change

**Figure 1:**
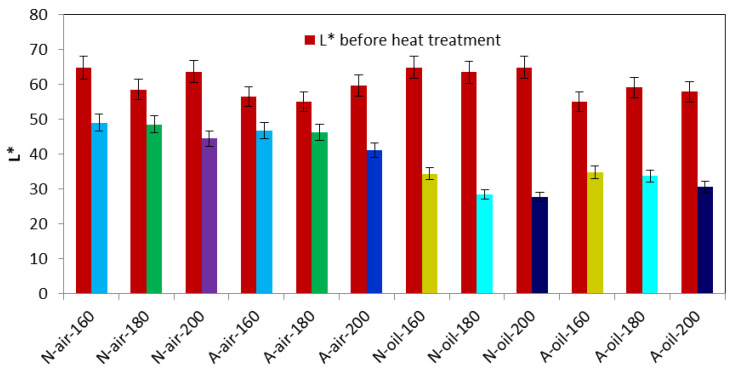
The influence of heat treatment under air or palm oil on Afrormosia and Newtonia on the $L^{*}$ parameter.

The results of color measurements of Newtonia and Afrormosia wood after heat treatment under air and palm oil as well as the related statistical analyses are shown in Table 2. From the results, it can be seen that ΔE* and ΔL* are substantially equal in absolute value, whether for heat treatment in air or in palm oil, and this remains true regardless of the treatment temperature considered. In other words, statistically speaking, the average values of a* and b* were not modified by the heat treatment in air of Newtonia and Afrormosia wood. Therefore, these woods practically kept their yellow and red color after the treatment. It can therefore indicate that it is L* the main parameter that determines the evolution of the color during wood heat treatment [36]. Otherwise, L* significantly decreased with temperature for both Afrormosia and Newtonia wood treated with air and palm oil (Figure 1). This decrease of the parameter L* was greater for Afrormosia in the case of heat treatment under air and more pronounced for Newtonia for treatment carried out under palm oil. This means that the heat treatment of Newtonia and Afrormosia wood increases their dark coloration, and this increase is proportional to the treatment temperature [40]. In other words, Afrormosia and Newtonia wood treated under air or palm oil became darker after treatment. Čabalová [44], having obtained the same results, demonstrated that this observation is related to the fact that the lignin macromolecules degrade with the treatment temperature, producing extractives that give the dark color to the wood. Sikora [38] confirmed this result by showing that the amount of extractives in the wood increases with the treatment temperature.

**Figure 2:**
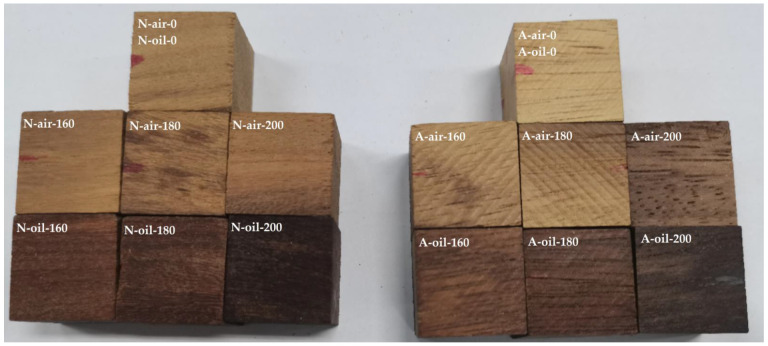
Wood samples before and after heat treatment.

In addition, we found that the darker shade was more pronounced for the palm oil heat treated wood (Figure 2). This is probably caused by the caramelization of sugars resulting from the degradation of hemicelluloses by palm oil, which largely depends on the treatment temperature [45]. Additionally, Afrormosia heat-treated under air blackened more than Newtonia treated under the same conditions. This tendency was reversed under heat treatment conditions with palm oil. The first case is justified by the fact that Afrormosia is naturally darker than Newtonia, which is characterized by the lower values of L* in Afrormosia than in Newtonia before treatment. The second case suggests that the caramelization of sugars resulting from the hydrolysis of hemicelluloses is more accentuated in Newtonia than in Afrormosia [26]. We have also noticed that the samples in the palm oil medium were blackened more than that in the air medium. This phenomenon can be explained by the auto-oxidation of unsaturated fatty acids and their derivatives from palm oil [11]. In sum, heat treatment under air and palm oil makes the wood blacker (Figure 1).

### 3.2. Weight Change

**Figure 3:**
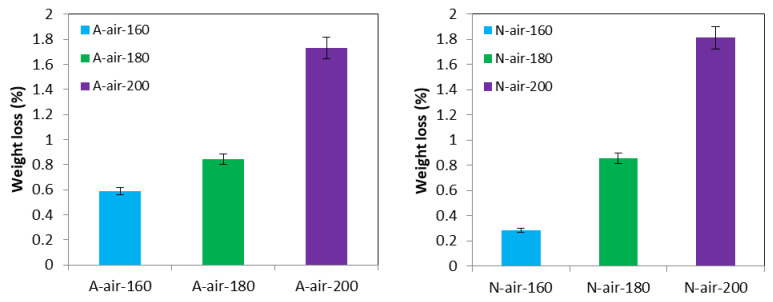
Weight loss of Afrormosia and Newtonia heat-treated under air.

**Figure 4:**
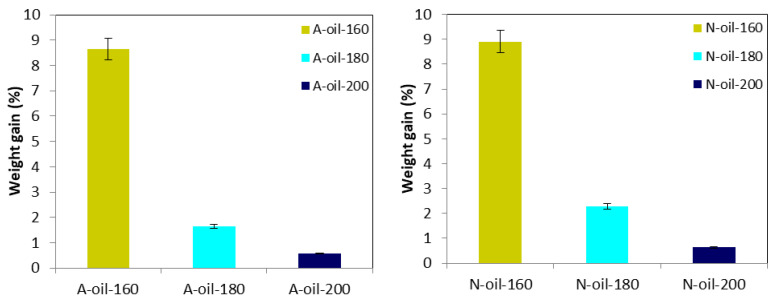
Weight percentage gain of Afrormosia and Newtonia heat-treated under palm oil.

The weight changes occurred during Afrormosia and Newtonia heat treatment are presented in Figure 3 and Figure 4. The loss in mass was only observable for the cases of thermal treatment under air conditions. As shown in Figure 3, the weight loss increased with the treatment temperature regardless of the species of wood, mainly due to the decrease with temperature, of the quantity of polysaccharides related mainly to the degradation of hemicelluloses [38]. Čabalová [44] goes further, stating that it is the degradation of Xylose, D-galactose, L-arabinose and D-mannose type polysaccharides that are responsible for the loss of wood mass during heat treatment. Moreover, at 200 °C, the three previously mentioned polysaccharides are completely degraded [38,46], which explains why we found in our study that the maximum losses was observed for the heat treatments carried out at the temperature of 200 °C and reached 1.81% and 1.71%, respectively, for Newtonia and Afrormosia.

The weight gain of samples treated in palm oil provides information on the mass of palm oil absorbed by the wood as well as the degradation of wood components during the heat treatment. Figure 4 presents the percentages of weight gains of Afrormosia and Newtonia wood heat-treated under palm oil as a function of the temperature. It can be seen that Newtonia tended to absorb more palm oil than Afrormosia. Moreover, with the more serious damage of wood hemicelluloses occurred at high temperature, the weight percentage gain also decreased with the increase of treatment temperature, regardless of the species of wood [23,43].

The density evolution of Afrormosia and Newtonia wood is presented in Table 3. From the said table, we observe that the heat treatment under air of Afrormosia and Newtonia woods reduced the density proportionally to the treatment temperature [17], contrary to the heat treatment with oil which rather increases the density. However, this increase in density decreased with treatment temperature caused by the degradation of hemicelluloses, which induces a loss in mass of the wood [24]. The most significant increase in density was observed in Afrormosia, while the most significant reduction in density was noticed in Newtonia.

### 3.3. Swelling

**Figure 5:**
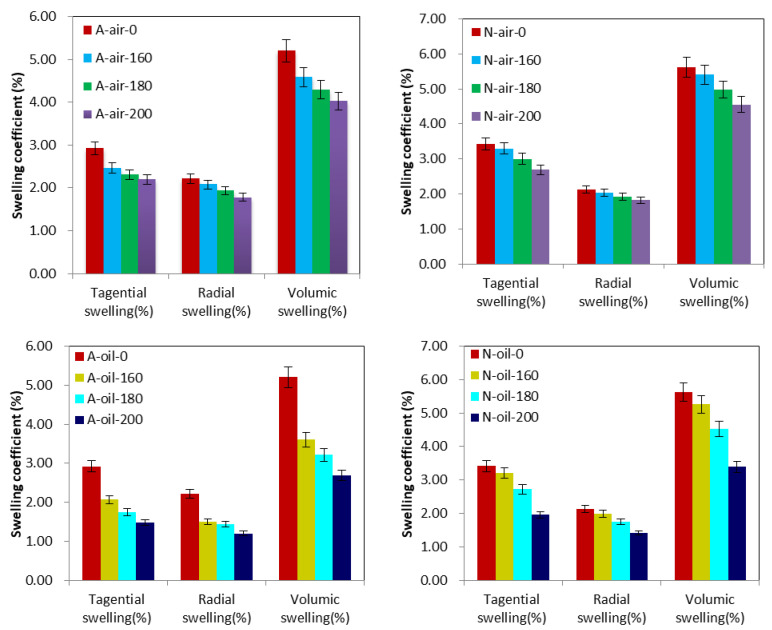
Swelling coefficients of Afrormosia and Newtonia heat-treated under air or palm oil.

Compared to the swelling of the control sample, the tangential swelling of Afrormosia heat-treated under air at 160 °C, 180 °C and 200 °C decreased by 15.79%, 21.17% and 24.59%, respectively, while its radial swelling under the same treatment conditions decreased by 6.44%, 12.62% and 19.58%, respectively. The heat treatment under palm oil has a greater influence on the improvement of the dimensional stability of Afrormosia wood. Indeed, treated under palm oil at 160 °C, 180 °C and 200 °C, Afrormosia wood underwent a respective decrease of its tangential swelling of 29%, 40% and 49.34% and also of its radial swelling of the order of 32.43%, 34.98% and 45.88%. We also observed a dimensional stabilization of Newtonia wood during the different heat treatments. In fact, its tangential swelling coefficient was lowered by 3.58% and 12.16% for the heat treatments carried out in air at temperatures of 160 °C and 180 °C, and by 21.32% at 200 °C, while its radial swelling for the same treatment at 160 °C, 180 °C and 200 °C was lowered by 4.13%, 9.97% and 14.77%, respectively. A similar observation was made for Newtonia wood treated with palm oil, since during this treatment at 160 °C, 180 °C and 200 °C the tangential swelling and radial swelling of Newtonia wood were, respectively, reduced by 6.05%, 20.37%, 42.85% and 6.79%, 17.82%, 33.63% (Figure 5).

The dimensional stability of the wood can also be evaluated using the ASE parameter. In fact, based on the definition of ASE (Equation (10)), it is easy to notice that this parameter is directly related to the volumetric swelling of the wood and allows for estimating the difference between the volume of the untreated wood after water absorption and the volume of the treated wood after water absorption in relation to the difference between this last volume and the anhydrous volume of the wood. In other words, the less the volume of heat-treated wood varies after water absorption, the higher the ASE [17]. Moreover, this is what we observed for heat treatment under air and oil of Afrormosia and Newtonia wood because, regardless of the wood species and type of treatment, the ASE of these woods increased proportionally with temperature and was inversely proportional to the volumic swelling (Table 4). Furthermore, we observed a higher ASE for Afrormosia and for samples heat-treated with palm oil (Table 4).

It is therefore clear that wood swelling is closely related to wood direction, treatment temperature and wood species. Afrormosia and Newtonia, like most woods, have less dimensional stability in the tangential direction than in the radial direction when absorbing water due to increased environmental relative humidity. Heat treatment with air or palm oil reduces the tendency for dimensional changes in the tangential and radial directions in proportion to the increase in processing temperature. In addition, swelling is mainly due to the increase in the distance between cellulose microfibrils in the wood cell wall caused by absorbed water [47]; thus, it seems that this distance is greater in Newtonia wood than in Afrormosia wood. This could explain why Afrormosia dimensions were better stabilized than Newtonia during heat treatment. The improvement in swelling observed for both air- and oil-treated samples is due to a decrease in the hydroxyl groups of the less thermally stable hemicelluloses having active sorption sites available in the wood cell walls. This improvement is also due to a reorganization of the chemical structure of the less ordered amorphous cellulose leading to a more ordered and hydromorphic crystalline cellulose, as this leads to a further reduction of available sorption sites [48]. Finally, the improvement in swelling can also be explained by crosslinking caused by the polycondensation reactions in lignin [26]. We found that heat treatment under palm oil stabilizes the wood better than heat treatment under air. This is explained by the fact that in heat treatment with palm oil, oil absorption is accompanied by oil deposits in the cell wall and this leads to the formation of a protective layer on the wood against water [26]. However, the oil absorbed by the wood is much less important for swelling than for water absorption [27].

### 3.4. Moisture Absorption (MA)

**Figure 6:**
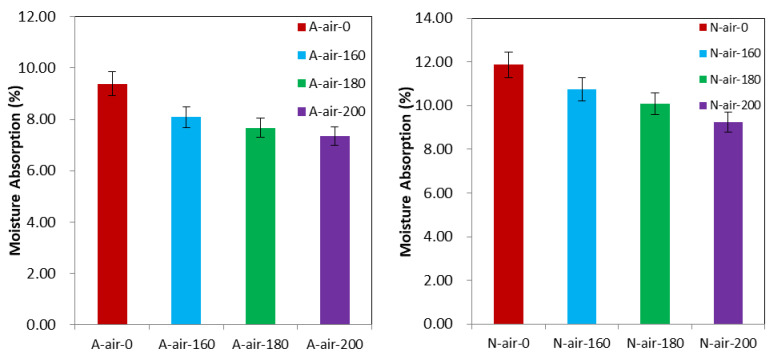
Moisture absorption of Afrormosia and Newtonia heat treated under air.

**Figure 7:**
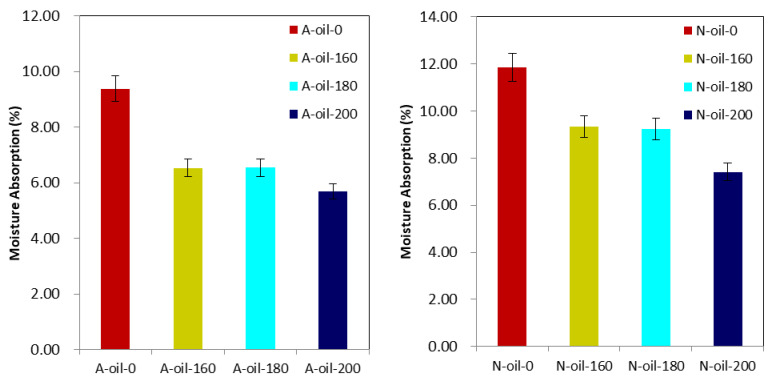
Moisture absorption of Afrormosia and Newtonia heat treated under palm oil.

Besides the effects on dimensional stability, heat treatment under air or palm oil also has a great influence on the moisture absorption of Afrormosia and Newtonia wood and therefore on their hygroscopicity. As shown in Figure 6 and Figure 7 (The difference between mean value before treatment and mean value after treatment is statistically significant for all the batches in Figure 6 and Figure 7), the reduction in water absorption of wood was proportional to the increase of treatment temperature. Thus, the maximum declines of the moisture absorption were observed in the treatments carried out at 200 °C. Compared with Newtonia, this decrease was greater for Afrormosia regardless of the treatment medium. Specifically, the heat treatments of Afrormosia samples in the air at 160 °C, 180 °C and 200 °C diminished the water absorption by 13.80%, 18.19%, and 21.67%, respectively, while those treatments for the same wood in palm oil decreased its water absorption by 30.34%, 30.75%, and 39.40%, respectively.

Similarly, the heat treatment of Newtonia wood in air at 160 °C, 180 °C and 200 °C lowered the water absorption by 9.36%, 14.92%, and 22.12%, respectively, while the heat treatment of the said wood under palm oil decreased its water absorption by 21.32%, 22.14%, and 37.48%, respectively. The moisture absorption capacity was therefore reduced with the increasing heat treatment temperature. The water absorption of wood is strongly linked to the availability of the hydroxyl groups of the polymers cellulose, hemicellulose and lignin contained in the cell wall [49]. The heat treatment, here implemented at high temperature, leads to the irreversible degradation of hemicelluloses [33] and, by extension, of the adsorption sites of water molecules, which accounts for the improvement of hygroscopicity wood. In addition, palm oil improves the moisture absorption of wood more effectively than air. This is because palm oil, unlike air, has a high number of unsaturated acids, which are characterized by the iodine value [50]. These unsaturated fatty acids bond with hydroxyl groups, forming an elastic film on the surface of the wood, which improves the hygroscopicity of the wood [51].

### 3.5. FTIR Analysis

**Figure 8:**
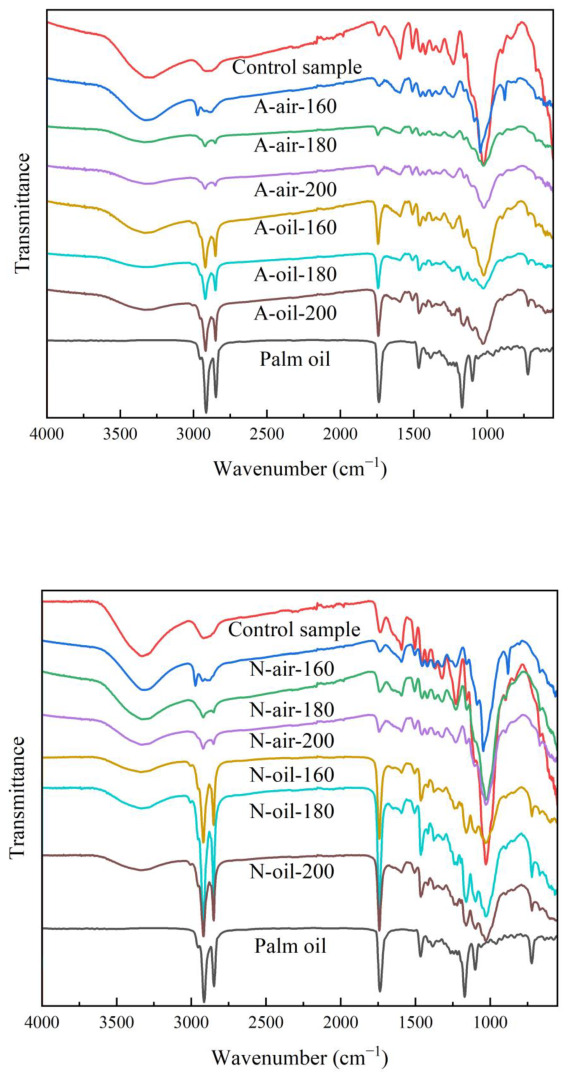
FTIR spectra of Newtonia and Afrormosia heat-treated under air or palm oil.

The analysis of the FTIR spectra was applied to all the different samples and was performed between the wavelengths of 4000 cm^−1^ and 400 cm^−1^. Regardless of the wood species and the treatment temperature, the general shape of the spectra remained similar when treated with the same heat transfer fluid (Figure 8). Around 3300 cm^−1^, we identified a broad band of OH stretching vibration in air and palm oil heat treatments [3]. This band decreased with increasing treatment temperature regardless of the heat transfer fluid used, indicating a decrease in OH groups in the wood samples. In other words, this means that heat treatment with air or palm oil reduced the hygroscopic capacity of Afrormosia and Newtonia wood and that this reduction was directly related to temperature and did not necessarily depend on the treatment medium, thus highlighting the degradation of hemicelluloses [52]. From 1260 to 1460 cm^−1^, medium intensity bands were observed, reflecting the vibrational stretching of hydroxyl bonds of primary, secondary, tertiary and aromatic alcohols. These bands were more accentuated for samples treated with palm oil and the intensity of these bands also decreased with increasing temperature. On the other hand, the broad band of high intensity with valence vibration located between 1030 cm^−1^ and 1050 cm^−1^ show the C-OH bonds of alcohols, the C-O bonds of ethers and the β-O-4 linkages in lignin. We found that this band decreases for treatment with temperatures below 200 °C but increases for wood treated at 200 °C. This increase suggests the generation of primary alcohols and likely results from the degradation of cellulose and hemicelluloses [25]. In addition, it also suggests the cleavage of β-O-4 bonds and the cleavage of methoxylates from lignin. Similar results have been found by Sikora [38]. We also identified a broad band of high intensity located between 2850 cm^−1^ and 2900 cm^−1^ that highlights the presence of C-H bonds of aldehydes [52]. The intensity of this band was stronger for the samples treated under palm oil. This is probably due to the use of palm oil. The fine band of high intensity observed around wavelength 1740 cm^−1^ of the wood samples treated with palm oil reflects the modification in carbohydrates (in hemicelluloses) by oxidation, of the content of C=O bonds of carboxylic acids due to palm oil [48,52,53]. The band at 1505 cm^−1^, which highlights the vibrational stretching of aromatic skeletal C=C in lignin, is more accentuated for palm oil heat treatment, implying that this treatment degrades the wood lignin more than air heat treatment. Indeed, methoxyl groups and syringyl units degrade faster than guaiacyl at high temperatures. In other words, palm oil at high temperature, more than air, favors the cleavage of methoxyl groups or the loss of syringyl units [38].

### 3.6. Comparison on the Efficiency (%) of Heat Treatment under Air and Heat Treatment under Palm Oil

Table 5 presents the comparison on the efficiency (%) of heat treatment under air and heat treatment under palm oil. In general, the heat treatment under palm oil better stabilizes the dimensions of the wood and results in more of a reduction in the water absorption of the said material compared to the heat treatment of the wood under air. However, it further blackens the wood and increases its mass contrary to the heat treatment under air, which rather reduces the mass of the wood.

## 4. Conclusions

Heat treatments in air and palm oil are both common and effective methods for dimensional stabilization and hygroscopicity improvement of Afrormosia and Newtonia wood. Indeed, the tangential and radial swelling coefficient for heat treatment with the air of Afrormosia wood at 200 °C were respectively reduced by 24.59% and 19.58%, while these reduction were 21.32% and 14.80% for Newtonia. The loss in mass was only observable for the cases of thermal treatment under air conditions. The maximum losses were observed for the heat treatments carried out at the temperature of 200 °C and reach 1.81% and 1.71%, respectively, for Newtonia and Afrormosia. The heat treatments of Afrormosia and Newtonia samples under air at 200 °C diminished the water absorption by 21.67% and 22.12%. The heat treatment under palm oil further improves the stability and hygroscopicity of the wood. In fact, a decrease of Afrormosia tangential and radial swelling coefficient at 200 °C by 49.34 %, 45.88% was observed, while a reduction of Newtonia tangential and radial swelling coefficient at the same temperature, by 42.85% and 33.63% was noticed. The water absorption of Afrormosia and Newtonia heat-treated under palm oil at 200 °C was reduced, respectively, by 39.40% and 37.49%. In addition, the samples of wood treated under palm oil conditions were darker than those treated under air conditions. The FTIR analysis showed that regardless of the wood species and heat transfer medium, the decrease of the band around the wavenumber of 3300 cm^−1^ indicates the reduction of hydroxyl groups, which leads to the improvement of dimensional stability and hygroscopicity of Afrormosia and Newtonia wood, resulting mainly from the degradation of hemicelluloses due to the increase in temperature. In summary, palm oil heat treatment has many advantages over air heat treatment and may have more potential for effective modification of wood in the future.

## Figures and Tables

**Table 1:** Labelling of samples.

Wood Species	Heat Treatment Medium	Control Samples	160 °C	180 °C	200 °C
Afrormosia	air	A-air-0	A-air-160	A-air-180	A-air-200
Afrormosia	Palm oil	A-oil-0	A-oil-160	A-oil-180	A-oil-200
Newtonia	air	N-air-0	N-air-160	N-air-180	N-air-200
Newtonia	Palm oil	N-oil-0	N-oil-160	N-oil-180	N-oil-200

**Table 2:** The influence of heat treatment under air or palm oil on Afrormosia and Newtonia color.

**Samples**	**Before Treatment**			**After Treatment**			ΔL*	Δa*	Δb*	ΔE*
	$L^{*}$	$a^{*}$	$b^{*}$	$L^{*}$	$a^{*}$	$b^{*}$				
A-air-160	56.45	12.98 ^p^	24.2 ^p^	46.80	13.19 ^p^	19.77 ^p^	−9.65	0.21	−4.43	10.62
A-air-180	55.11	11.52 ^p^	22.72 ^p^	46.23	12.29 ^p^	21.29 ^p^	−8.88	0.77	−1.43	9.03
A-air-200	59.69	11.72 ^p^	23.69 ^p^	41.2	12.18 ^p^	18.76 ^p^	−18.49	0.46	−4.93	19.14
N-air-160	64.74	9.29 ^p^	16.42 ^p^	49.01	10.12 ^p^	15.34 ^p^	−15.73	0.83	−1.08	15.79
N-air-180	58.53	10.09 ^p^	17.47 ^p^	48.52	10.58 ^p^	16.33 ^p^	−10.01	0.49	−1.14	10.09
N-air-200	63.62	8.80 ^p^	17.78 ^p^	44.45	7.57 ^p^	10.59 ^p^	−19.17	−1.23	−7.19	20.51
A-oil-160	55.03	12.54	24.82	34.77	15.35	16.11	−20.26	2.81	−8.71	22.23
A-oil-180	59.12	11.92	24.39	33.75	13.21	14.57	−25.37	1.29	−9.82	27.23
A-oil-200	57.93	11.65	22.77	30.66	8.64	8.71	−27.27	−3.01	−14.06	30.83
N-oil-160	64.89	9.65 ^p^	17.15	34.33	11.94 ^p^	13.36	−30.56	2.29	−3.79	30.88
N-oil-180	63.50	10.19 ^p^	18.46	28.45	7.24 ^p^	6.55	−35.05	−2.95	−11.91	37.14
N-oil-200	64.89	8.44	15.65	27.78	6.83	4.80	−37.11	−1.61	−10.85	38.70

^p^: Mean value before treatment and mean value after treatment are statistically identical.

**Table 3:** The influence of heat treatment under air or palm oil on Afrormosia and Newtonia density.

Sample Handling	Density	Density Change (%) Percentage (%)
N-air-0	0.719	-
N-air-160	0.700	−2.59
N-air-180	0.690	−3.98
N-air-200	0.676	−6.00
A-air-0	0.786	-
A-air-160	0.780	−0.70
A-air-180	0.764	−2.81
A-air-200	0.759	−3.43
N-oil-0	0.719	-
N-oil-160	0.747	3.94
N-oil-180	0.742	3.22
N-oil-200	0.733	1.98
A-oil-0	0.777	-
A-oil-160	0.874	12.46
A-oil-180	0.819	5.46
A-oil-200	0.810	4.30

**Table 4:** Swelling coefficients and anti-swelling efficacy values of Afrormosia and Newtonia heat-treated under air or palm oil.

Sample Handling *	Tangential Swelling (%)	Radial Swelling (%)	Volumic Swelling (%)	Anti-Swelling Efficacy (%)
A-air-0	2.92	2.22	5.2	-
A-air-160	2.46	2.07	4.58	11.87
A-air-180	2.30	1.94	4.28	17.65
A-air-200	2.20	1.78	4.02	22.64
N-air-0	3.42	2.13	5.62	-
N-air-160	3.30	2.04	5.41	3.84
N-air-180	3.00	1.92	4.98	11.44
N-air-200	2.69	1.82	4.56	18.97
A-oil-160	2.07	1.50	3.60	30.76
A-oil-180	1.75	1.44	3.22	38.13
A-oil-200	1.48	1.20	2.70	48.15
N-oil-160	3.21	1.99	5.26	6.39
N-oil-180	2.72	1.75	4.52	19.57
N-oil-200	1.95	1.42	3.40	39.59

* The difference between mean value before treatment and mean value after treatment is statistically significant for all the batches in Table 4.

**Table 5:** Comparison on the efficiency (%) of heat treatment under air and heat treatment under palm oil.

Heat Treatment Medium	Samples	Efficiency (%) Based on Control Samples					
		a*	b*	L*	Radial Swelling	Tangential Swelling	Moisture Absorption
Heat treatment under air	A-air-160	1.62	−18.31	−17.09	−6.44	−15.79	−13.80
	A-air-180	6.68	−6.29	−16.11	−12.62	−21.17	−18.19
	A-air-200	3.92	−20.81	−30.98	−19.58	−24.59	−21.67
	N-air-160	8.93	−6.58	−24.30	−4.13	−3.58	−9.36
	N-air-180	4.86	−6.53	−17.10	−9.97	−12.16	−14.92
	N-air-200	−13.98	−40.44	−30.13	−14.77	−21.32	−22.12
Heat treatment under palm oil	A-oil-160	22.41	−35.09	−36.82	−32.43	−29.03	−30.34
	A-oil-180	10.82	−40.26	−42.91	−34.97	−40.03	−30.75
	A-oil-200	−25.84	−61.75	−47.07	−45.88	−49.34	−39.39
	N-oil-160	23.73	−22.10	−47.10	−6.79	−6.05	−21.32
	N-oil-180	−28.95	−64.52	−55.20	−17.82	−20.37	−22.14
	N-oil-200	−19.08	−69.33	−57.19	−33.63	−42.85	−37.49

The minus sign “−” means that the heat treatment has reduced the value of the measured parameter.

## Data Availability

The data that allowed for the writing of this article are available from the Wood Science and Technology department/ School of Material Science and Technology of the Beijing Forestry University.
